# Health impact and cost-effectiveness of HIV testing, linkage, and early antiretroviral treatment in the Botswana Combination Prevention Project

**DOI:** 10.1097/QAI.0000000000002996

**Published:** 2022-04-14

**Authors:** Stephen C. Resch, Julia H. A. Foote, Kathleen E. Wirth, Arielle Lasry, Justine A. Scott, Janet Moore, Fatma M. Shebl, Tendani Gaolathe, Mary K. Feser, Refeletswe Lebelonyane, Emily P. Hyle, Mompati O. Mmalane, Pamela Bachanas, Liyang Yu, Joseph M. Makhema, Molly Pretorius Holme, Max Essex, Mary Grace Alwano, Shahin Lockman, Kenneth A. Freedberg

**Affiliations:** 1 Department of Health Policy and Management, Harvard T. H. Chan School of Public Health, 677 Huntington Avenue, Kresge 3^rd^ & 4^th^ Floors, Boston, MA 02115, USA; 2 Medical Practice Evaluation Center, Department of Medicine, Massachusetts General Hospital, 100 Cambridge Street, 16^th^ Floor, Boston, MA 02114, USA; 3 Department of Epidemiology, Harvard T. H. Chan School of Public Health, 677 Huntington Avenue, Boston, MA 02115, USA; 4 Department of Immunology and Infectious Diseases, Harvard T. H. Chan School of Public Health, 651 Huntington Avenue, Boston, MA 02115, USA; 5 Division of Global HIV & TB, Center for Global Health, Centers for Disease Control and Prevention, 1600 Clifton Road, Atlanta, GA 30329, USA; 6 Harvard Medical School, 25 Shattuck Street, Boston, MA 02115, USA; 7 Botswana-Harvard AIDS Institute Partnership, Princess Marina Hospital, Plot No. 1836, Northring Road, Gaborone, Botswana; 8 Botswana Ministry of Health and Wellness, Plot 54609, 24 Amos Street, Government Enclave, Gaborone, Botswana; 9 Division of Infectious Diseases, Massachusetts General Hospital, 55 Fruit Street, Boston, MA 02144, USA; 10 Harvard University Center for AIDS Research, 42 Church Street, Cambridge, MA 02138, USA; 11 Centers for Disease Control and Prevention, Gaborone, Botswana; 12 Division of Infectious Diseases, Brigham and Women’s Hospital, 45 Francis Street, 2^nd^ Floor, Boston, MA 02115, USA; 13 Division of General Internal Medicine, Massachusetts General Hospital, 50 Staniford Street, 9^th^ Floor, Boston, MA 02114, USA

**Keywords:** Cost-effectiveness, Combination prevention, HIV prevention, Modeling, Economic analysis, Botswana

## Abstract

**Background:**

The Botswana Combination Prevention Project tested the impact of combination prevention (CP) on HIV incidence in a community-randomized trial. Each trial arm had ~55,000 people, 26% HIV prevalence, and 72% baseline ART coverage. Results showed intensive testing and linkage campaigns, expanded antiretroviral treatment (ART), and voluntary male medical circumcision (VMMC) referrals increased coverage and decreased incidence over ~29 months follow-up. We projected lifetime clinical impact and cost-effectiveness of CP in this population.

**Setting:**

Rural and peri-urban communities in Botswana.

**Methods:**

We used the Cost-Effectiveness of Preventing AIDS Complications (CEPAC) model to estimate lifetime health impact and cost of 1) earlier ART initiation, and 2) averting an HIV infection, which we applied to incremental ART initiations and averted infections calculated from trial data. We determined the incremental cost-effectiveness ratio (ICER, US$/QALY) for CP vs. standard of care.

**Results:**

In CP, 1,418 additional people with HIV initiated ART and an additional 304 infections were averted. For each additional person started on ART, life expectancy increased 0.90 QALYs and care costs increased by $869. For each infection averted, life expectancy increased 2.43 QALYs with $9,200 in care costs saved. With CP, an additional $1.7 million were spent on prevention and $1.2 million on earlier treatment. These costs were mostly offset by decreased care costs from averted infections, resulting in an ICER of $79 per QALY.

**Conclusions:**

Enhanced HIV testing, linkage, and early ART initiation improves life expectancy, reduces transmission, and can be cost-effective or cost-saving in settings like Botswana.

## INTRODUCTION

With an estimated 20% of the adult population living with HIV, Botswana has the third highest HIV prevalence globally.^1^ By 2015, Botswana had nearly achieved the UNAIDS “90-90-90” targets for HIV diagnoses, treatment, and viral suppression.^2,3^ Even so, annual HIV incidence among adults remained high at 1.3%.^3,4^

The Botswana Combination Prevention Project (BCPP, also known as the Ya Tsie trial) was a cluster-randomized trial designed to examine whether a combination of HIV prevention measures (CP) could reduce HIV incidence, compared to standard of care (SOC).^5^ Prevention measures in CP included activities approximating universal test- and-treat (intensive HIV testing campaigns, improved linkage to care, and expanded antiretroviral treatment (ART) eligibility) and increased referrals for voluntary medical male circumcision (VMMC).

In the trial, annual HIV incidence was 30% lower with CP than in the SOC arm over a 29-month follow-up period.^5^ The trial also reported increases in HIV status knowledge, treatment coverage, viral suppression, and male circumcision coverage in communities exposed to the CP package compared with the SOC.^5,6^ Around the same time, two other international trials, PopART and SEARCH, also showed that universal test-and-treat led to improved HIV outcomes and decreased transmission.^7,8^

Our objective was to estimate the cost-effectiveness of the combination prevention package in the BCPP trial.

## METHODS

### BCPP Trial Overview

BCPP was a pair-matched, cluster-randomized clinical trial from October 2013 to June 2018, in 30 rural and peri-urban communities; it was designed to determine whether implementation of CP could reduce HIV incidence at the community level.^5^ Fifteen matched pairs of communities were randomized between the CP arm and the SOC arm. Each arm had a population (age 16–64) of ~55,000 with HIV prevalence 26% and ART coverage 72% at baseline. SOC communities received limited technical support at local HIV clinics. Both SOC and CP communities received HIV testing in a random sample of 20% of households (with clinic referral of persons with HIV) as part of a baseline household survey and annual follow-up surveys. In addition, CP communities received a combination of prevention activities including an intensive “saturation” campaign of door-to-door and mobile HIV testing and counseling, increased linkage-to-care efforts for those testing positive, and increased VMMC referrals for men testing negative. The CP arm offered expanded ART eligibility relative to SOC, though SOC changed over time with international guidelines (Supplement,http://links.lww.com/QAI/B842).

All trial participants provided written informed consent. Participants 16–17 years old provided written assent and written permission from their parents or guardians. The trial was approved by the institutional review boards at the Botswana Ministry of Health and Wellness and the US Centers for Disease Control and Prevention.

Indicators measured in a subset of three control and three intervention communities at baseline (random ~20% sample) and study completion (remaining ~80% of households) found an increase in ART coverage of 19 percentage points in intervention communities, compared to a ten-percentage point increase in control communities and a corresponding prevalence ratio of 1.12 (95% CI, 1.07 to 1.17).^6^ VMMC coverage in men aged 15–49 increased ten percentage points in intervention communities (30% to 40%) compared to two percentage points in control communities (33% to 35%) (prevalence ratio 1.26; 95% CI, 1.17 to 1.35).^6^ A 31% HIV incidence reduction (unadjusted incidence ratio, 0.69; 95% CI, 0.46 to 0.90, p = 0.09) was demonstrated in the intervention arm. Additional trial details have been published previously.^3,5^

### Modeling Analysis Overview

We used results from the BCPP trial to populate CEPAC, a computer microsimulation of HIV disease. Outside the model, we used trial data to estimate key outcomes which were used to scale model output: the incremental number of PWH started on ART and number of infections averted during the trial due to CP. We then populated CEPAC with trial data to estimate lifetime HIV-related care costs and quality-adjusted life expectancy for people in the CP and SOC arms.

**Figure 1.**
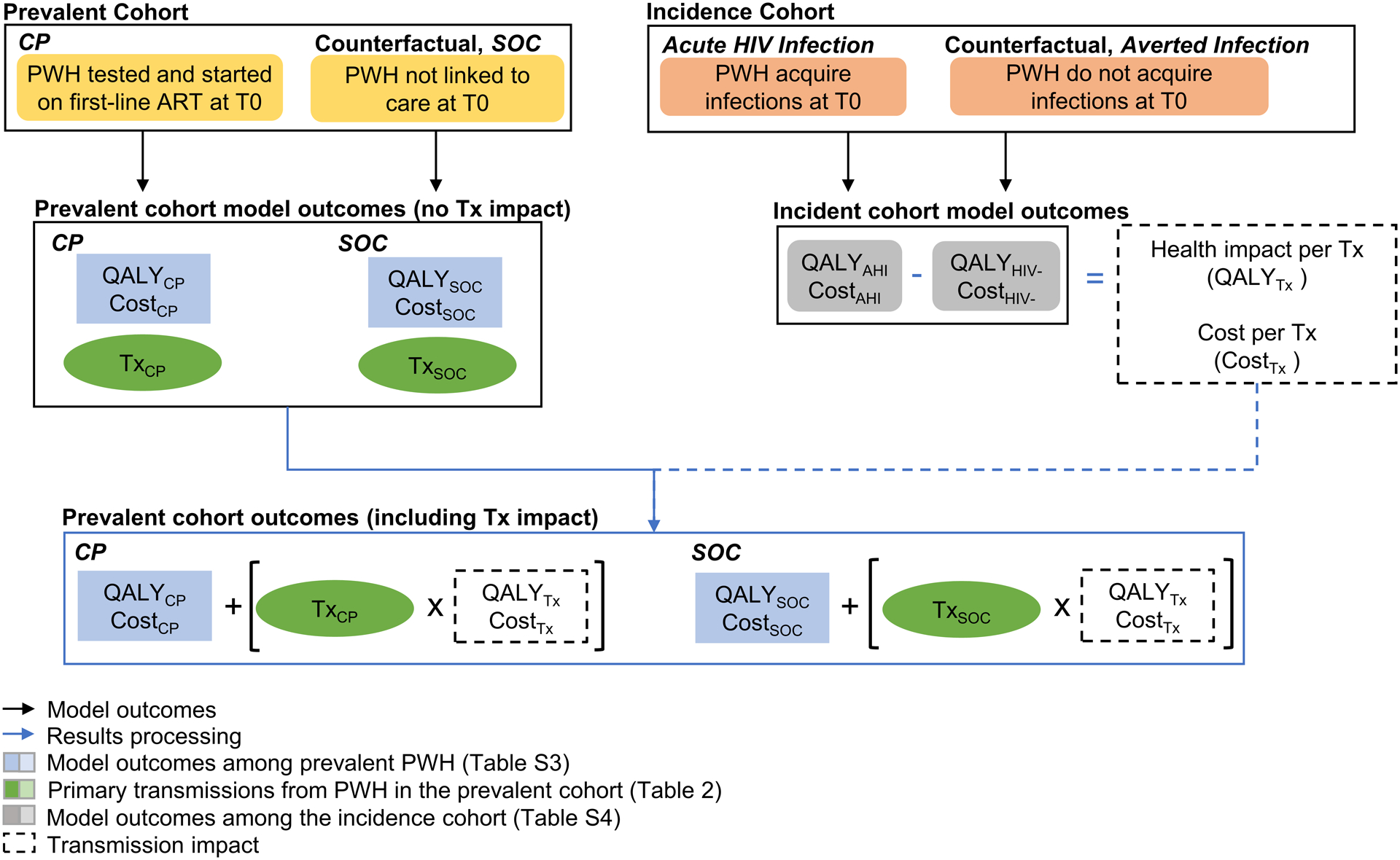
Overview of methods and model structure: cost-effectiveness of the Botswana Combination Prevention Project (BCPP). This figure outlines the CEPAC model runs used in the cost-effectiveness analysis of the Botswana Combination Prevention Project. Prevalent cohort: The CP subcohort represents PWH who were detected and started on first-line ART during the BCPP trial. The counterfactual SOC represents PWH who were not detected or linked to care through the trial (they may be linked through standard of care testing). Discounted quality-adjusted life years and costs among prevalent PWH excluding the impact of transmissions (QALY_CP_/ QALY_SOC_ and Cost_CP_/Cost_SOC_) are shown in blue. Primary transmissions from PWH in the prevalent cohort (Tx_CP_/Tx_SOC_) are shown in green. Incidence cohort: The *Acute HIV Infection (*AHI*) sub*cohort represents participants who acquired HIV during the BCPP trial and the counterfactual *Averted Infection* (HIV−) represents participants who did not aquire HIV during the trial. Model outcomes include discounted quality-adjusted life years and costs (QALY_AHI_/QALY_HIV−_ and Cost_AHI_/Cost_HIV−_) and are shown in grey. The lifetime difference in discounted quality-adjusted life-years and costs between the *Acute HIV* and *Averted Infection* cohorts represents the negative health impact (QALY_Tx_) and additional costs (Cost_Tx_) per transmission. To determine prevalent model outcomes including transmission impact, we multiply the impact per transmission (QALY_Tx_ and Cost_Tx_) by the number of first order transmissions in the CP and SOC subcohorts. We add the product to the discounted quality-adjusted life years (QALY_CP_/ QALY_SOC_) and costs (Cost_CP_/Cost_SOC_) for each subcohort. Prevalent cohort outcomes including transmission impact are found in Table 2. **CP**, combination prevention; **SOC**, standard of care; **PWH**, people with HIV; **ART**, antiretroviral therapy; **T0**, time zero (model iniation); **QALY**, quality-adjusted life-years; **Tx**, transmission; **AHI**, acute HIV infection; **HIV-**, no HIV infection.

Outcomes for each arm accounted for PWH started on ART and the people in whom infection was averted during the trial. We used CEPAC to estimate the additional infections that would be averted over a ten-year horizon after the trial due to the additional PWH started on ART. Therefore, the total health benefit comprises both quality-adjusted life years (QALYs) gained by PWH who started ART earlier and QALYs gained by people in whom infection was averted due to CP. Lifetime care costs for these groups were combined with the programmatic cost of the CP intervention to estimate the total cost (2019 US$). We calculated the incremental cost-effectiveness ratio (ICER) of CP vs. SOC by dividing the additional cost of CP by the additional QALYs generated. An overview of the approach is shown in Figure 1 (additional detail Figure S1).

### The CEPAC Model

CEPAC is a widely published and validated microsimulation of HIV infection, screening, disease progression, and treatment in resource-limited settings.^9,10^ Simulated patients are generated from user-defined distributions of sex, age, initial CD4 count, HIV RNA, and treatment adherence, then followed monthly from model entry until death. HIV diagnosis occurs via testing or presentation with an opportunistic infection (OI).

The model simulates probabilities of linkage to care, virologic suppression on ART, retention in care, and risk of OIs. ART is initiated according to strategy-specific CD4 count and viral load thresholds and the occurrence of user-defined primary and secondary OIs. When virally suppressed on ART, patients experience increasing CD4 counts. In the absence of treatment, patients experience a monthly decline in CD4 count and increased risk of OIs and mortality. Additional model details have been reported and are available at https://mpec.massgeneral.org/cepac-model/.^10,11^

### Estimation of Incremental PWH Starting ART

In the trial’s CP arm, 3,065 PWH were started on ART, 455 via the baseline survey and 2,610 via the testing campaign. Accounting for observed differences in linkage between the arms,^5^ we estimated that the baseline survey in the control arm resulted in 388 ART initiations. The trial did not collect data on HIV ‘background testing’ or ART enrollment occurring before trial activities. However, background testing is important since the testing campaign could ‘crowd out’ some background testing that would have otherwise occurred in the intervention arm. Case detection through background testing was estimated for each arm using trial data on ART coverage and case-finding associated with trial activities (Supplement,http://links.lww.com/QAI/B842).

### Incremental Lifetime Outcomes

We used the CEPAC model to project long-term differences in survival, onward transmission, and HIV-related care costs between CP and SOC among two cohorts. First, for CP we simulated a cohort representing additional PWH started on ART during the trial, with characteristics matching those observed in the group started on ART during the trial in terms of age, sex, CD4 count at ART start, and HIV RNA. For SOC, we simulated the same cohort under a counterfactual in which they were not started on ART during the trial. Second, for CP we simulated the cohort of people in whom infections were averted during the BCPP trial; characteristics matched those in both arms of the trial’s incidence cohort diagnosed with HIV during the trial. For SOC we modeled the counterfactual in which those people acquired HIV during the trial (Supplement Figure S1,http://links.lww.com/QAI/B842). Comparing the intervention to the counterfactual, we calculated the difference in lifetime QALY and cost outcomes for both cohorts. The simulation started at the end of the trial (T0 in Figure 1) and continued over a lifetime horizon.

### Cost-Effectiveness

We calculated the ICER for CP vs. SOC by dividing the difference in cost between the two strategies by the difference in QALYs. As a benchmark for cost-effectiveness, we compared the ICER for CP vs. SOC to a threshold of $3,981, equal to 0.5x Botswana’s 2019 annual *per capita* GDP.^12–14^ We report undiscounted health outcomes but use discounted health and cost outcomes (3% per year) for the cost-effectiveness analysis, as recommended by the Second Panel on Cost-Effectiveness in Health and Medicine.^15^

### Model Input Parameters

#### Cohort characteristics

Characteristics of the cohort of PWH who started ART due to the intervention reflected trial participants with HIV previously undiagnosed or not linked to care at trial start, measured during the baseline survey. Mean (±SD) initial age was 37y (±11y), 68% were female, and mean initial CD4 count was 449 cells/μL (±266 cells/ μL) (Table 1).

Characteristics of the cohort of persons in whom HIV was averted reflected study participants who tested negative at baseline and became infected during the trial. Mean age was 35y (±11y) and 67% were female. For those who eventually acquired HIV (projected after the trial period), mean initial CD4 count was 569 cells/μL (±226 cells/μL) at diagnosis.^5^

#### Transmissions

To project first-order transmissions averted after the trial period due to incremental ART initiation in prevalent PWH, we used model-based estimates of community viral load in conjunction with viral load-specific monthly transmission rates ranging from 0.00–9.03 transmissions/100 person-years (PY), with an acute transmission rate of 62.56 transmissions/100 PY.^16^

#### HIV incidence

In modeling the lifetime impact of averting an infection, the monthly probability of acquiring HIV after the trial period was from age-specific incidence rates observed in the trial’s CP intervention arm, ranging from 0.24 – 0.84/100 PY.^5,6^

#### Treatment efficacy and engagement in care

Data on adherence, ART efficacy, and engagement in care were from the BCPP trial. Average virologic suppression at 48 weeks across both adherence groups was 98%. For individuals in care, we modeled adherence-dependent loss-to-follow-up (LTFU) ranging from 0.23% monthly probability for those most adherent to 0.64% for those less adherent, resulting in about 7% of individuals experiencing loss to follow-up over two years—similar to rates observed in the trial population.^17^ Once lost to follow-up, individuals do not receive ART and experience the natural history of HIV. We assumed individuals return to care at a monthly probability of 1.0% (after 12 months out of care) or with an OI.

#### Natural history

We derived monthly probabilities of HIV-related mortality stratified by CD4 count, history of prior OI, and treatment status from mortality in the BCPP trial (Table S1). Using World Population Prospects and WHO data, we derived monthly non-AIDS-related mortality in Botswana by age and sex.^18,19^

#### Intervention cost

For cost outcomes, we first estimated the cost of delivering the CP intervention from an analysis of the BCPP testing campaign costs^20^ and program data (Supplement Table S2,http://links.lww.com/QAI/B842). In the CP arm, we applied the scaled $32.76/person cost to each person assessed through CP campaigns or the baseline survey. We added the cost of linkage counselors, background testing, and VMMC procedures. Each of the 15 intervention communities was assigned a cost of $13,110 for all linkage counseling activities throughout the trial period (Personal communication, Arielle Lasry). We used costs of $11.15/person for background testing and $116/VMMC procedure (Table S2).^21,22^ In the SOC arm, we applied the $32.76/person cost to each person tested through the baseline survey, since the door-to-door household testing was similar to the intervention arm testing campaign. Unit costs for background testing and VMMC were assumed the same as in the CP arm.

#### HIV-related care costs

We calculated HIV-related care costs by multiplying model-estimated resource utilization (e.g., outpatient visits and inpatient days) by unit costs. Annual first- and second-line ARV drug costs from the Clinton Health Access Initiative were $77.76 and $288.36.^23^ We used the Botswana consumer price index and the average 2019 exchange rate to convert all costs to 2019 U.S. dollars (Supplement Table S2,http://links.lww.com/QAI/B842).^24,25^

### Sensitivity Analysis

In sensitivity analysis, we considered uncertainty in main trial outcomes including infections averted during the trial, increase in ART coverage, and CP intervention costs. We also considered uncertainty in parameters used in the CEPAC model to simulate long-term outcomes after the trial including HIV care costs, monthly probability of receiving an HIV test, probability of linkage to ART if tested positive, and viral load-based monthly probabilities of transmitting HIV. We varied each parameter from 10% to as much as 300% of its base case value. For multi-way sensitivity analysis, we varied the incremental increase in ART coverage from 0.5 to 9.0 percentage points (base case: nine percentage points) and simultaneously adjusted the number of infections averted from 5% to 100% of the base case level, as ART coverage and number of infections averted are expected to be correlated. This range covers the lower bound of uncertainty intervals for main trial outcomes regarding infections averted and ART coverage.^5^ We simultaneously varied the incremental cost of the CP intervention from 100% to 250% the base case (base case: $1.7 million).

### Role of the Funding Source

The study sponsor had no role in study design, data collection, analysis, or interpretation, presentation of the findings, or the decision to submit the manuscript.

## RESULTS

### Base Case

Calibrating to observed differences in the increase in ART coverage between arms, we estimate that CP resulted in 1,418 more PWH started on ART than SOC (Table 1 and Supplement,http://links.lww.com/QAI/B842). During the trial, 262 infections were averted over 29 months in the intervention communities due to improved case-finding and linkage, earlier ART initiation, and VMMC coverage increases. Based on simulation of the lifetime of persons started on ART during the trial due to CP, we estimate an additional 42 infections averted in the ten years post-trial, for a total of 304 infections averted (Table 2).

Earlier HIV detection and ART initiation in the CP arm increased quality-adjusted life expectancy by 0.90 QALYs per person and lifetime HIV-related care costs by $869 per person started earlier on ART (Table S3). For people in whom an infection was averted, quality-adjusted life expectancy increased by 2.43 QALYs (from 17.03 to 19.46 QALYs) and $9,200 in HIV-related medical care costs were saved per person (from $9,970 to $730, Table S4).

Using CEPAC model results, we estimate that 2,086 discounted QALYs were generated by CP, split about evenly between the 1,418 PWH started earlier on ART and the 304 persons in whom an infection was averted.

**Figure 2.**
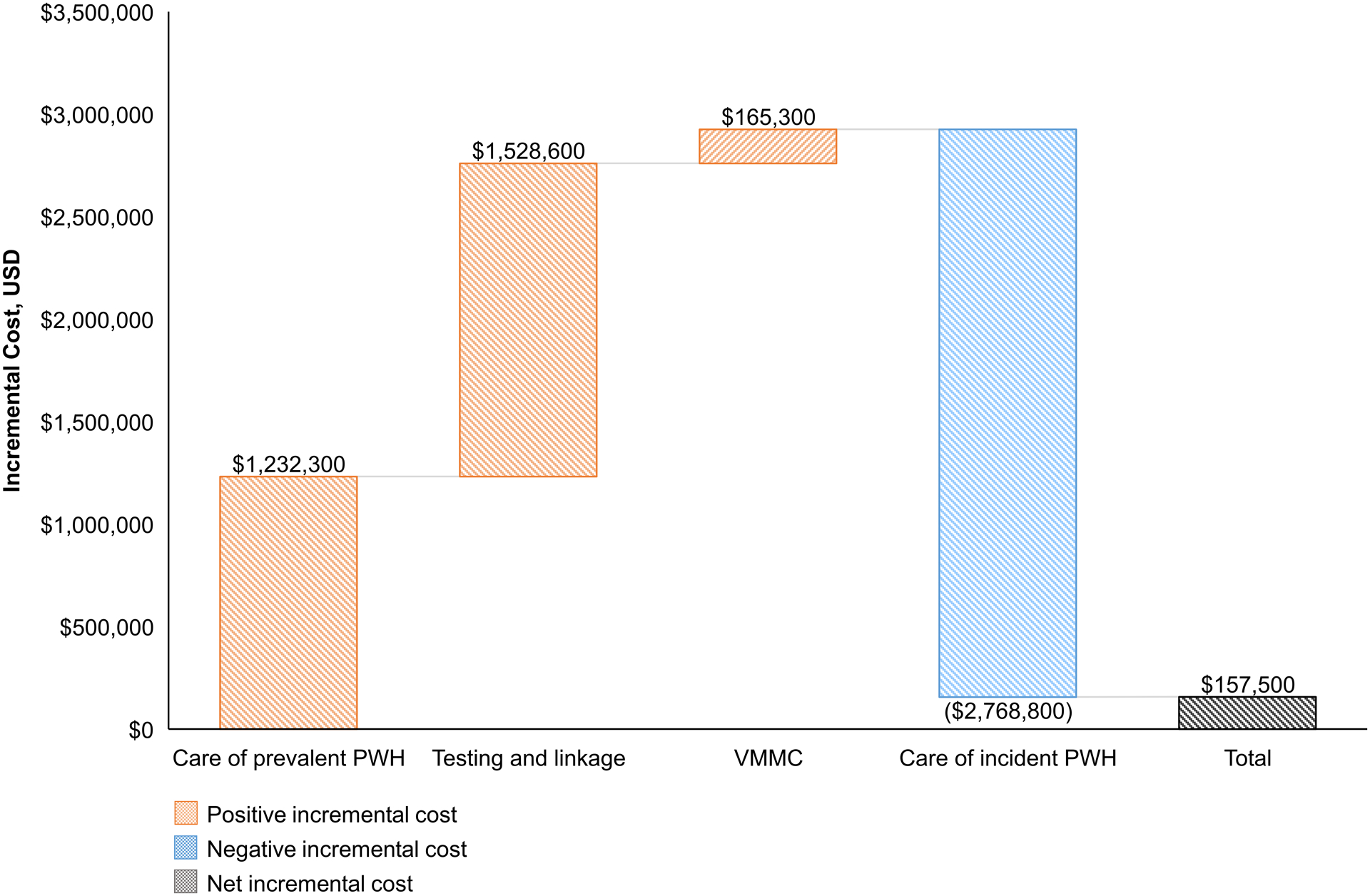
Cost breakdown of the incremental cost of the CP intervention. This waterfall chart reports the positive and negative incremental costs (y-axis) of each component (x-axis) of the CP intervention arm compared to the standard of care arm. Positive incremental costs are represented in orange, negative incremental costs in blue, and total or net incremental costs in grey. Exact cost values appear above or below the corresponding bar. **CP**, combination prevention; **SOC**, standard of care; **PWH**, people with HIV; **VMMC**, voluntary male medical circumcision.

Testing and linkage activities in the intervention arm, including those related to the baseline survey, cost $2.22 million. We estimated that another $111,000 was spent on background testing and $207,000 on 1,777 VMMC procedures in the intervention arm. In the standard of care arm, we estimate a cost of $208,000 for testing and $41,000 for VMMC related to the baseline survey, as well as $592,000 in background testing costs. Thus, the incremental total direct cost of CP activities was $1.69 million (Figure 2).

In addition to the incremental cost of delivering the intervention, we estimated the incremental cost of HIV care for the 1,418 PWH started on ART due to CP at $1.23 million. The $2.8 million in HIV care costs saved due to the 304 infections prevented offset most of the cost of the CP activities and consequent HIV care for PWH detected and started on ART, resulting in a net cost of $157,000 (Table 2 and Figure 2) and an ICER of $79 per QALY gained.

### Sensitivity Analysis

**Figure 3.**
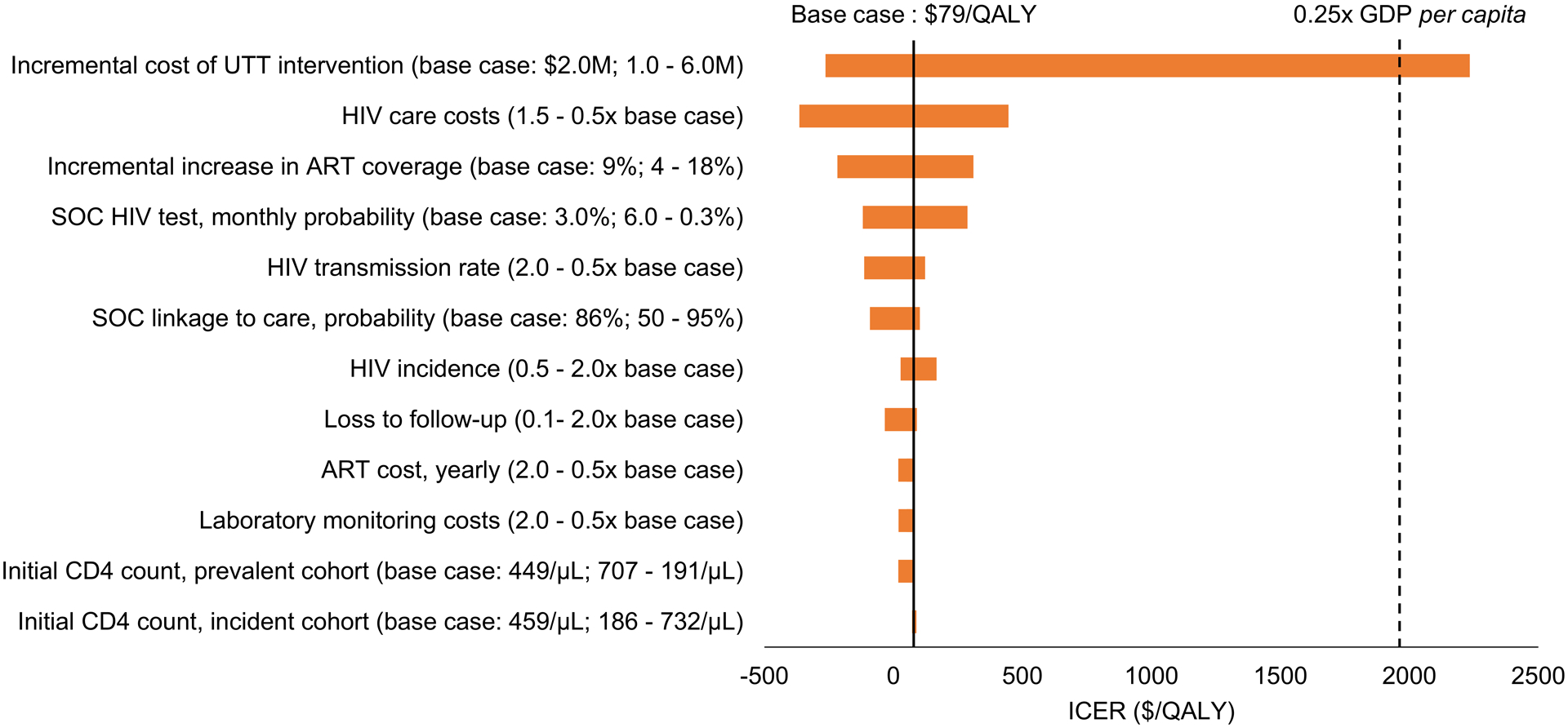
One-way sensitivity analyses on the cost-effectiveness ($/QALY) of CP compared to SOC in Botswana, including the impact of first-order HIV transmissions over ten years. This tornado diagram represents the ICERs (x-axis) for CP compared to SOC after input parameters (y-axis) were varied. The base case value for each input parameter is listed in parentheses before the semi-colon. The range across which we varied each parameter is listed after the semi-colon, with the value resulting in the lowest ICER before the hyphen and the value resulting in the highest ICER after the hyphen. The range of ICERs for each varied parameter is indicated by the horizontal bars. Longer horizontal bars indicate parameters to which the model results are most sensitive. The solid black line indicates the ICER for CP vs. SOC in the base case ($79/QALY). The dotted black line indicates 0.25x Botswana’s *per capita* GDP in 2019. **CP**, combination prevention; **SOC**, standard of care; **ART**, antiretroviral therapy; **ICER**, incremental cost-effectiveness ratio; **QALY**, quality-adjusted life year.

In univariate sensitivity analysis, the ICER for CP vs. SOC was consistently <0.5x Botswana’s annual *per capita* GDP. Other parameters used in simulating long-term impacts did not substantially affect the ICER when varied across wide ranges (Figure 3).

**Figure 4.**
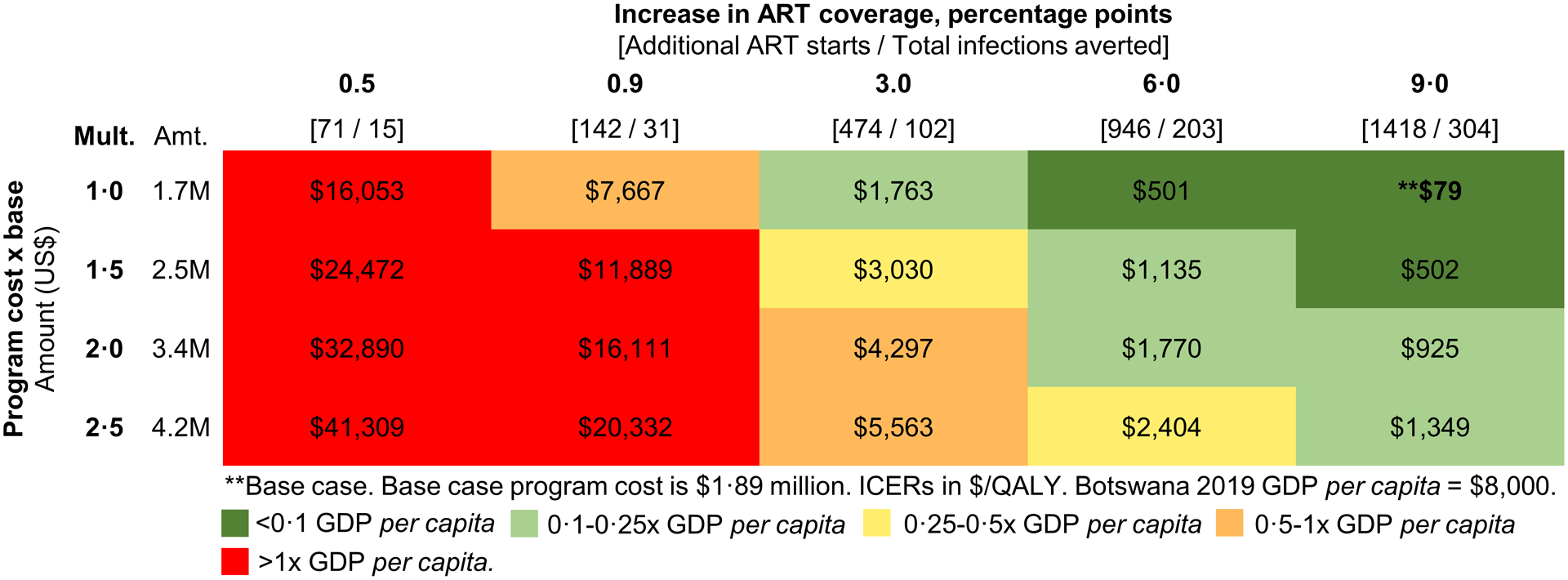
Two-way sensitivity analysis: Cost-effectiveness as a function of the incremental increase in ART coverage and the cost of the CP intervention. This heat map reports the ranges of incremental cost-effectiveness ratios of CP vs. SOC as a function of the two most influential parameters in Figure 3: incremental cost of the CP intervention (vertical axis) and incremental increase in ART coverage and infections averted (horizontal axis). Colors indicate the incremental cost-effectiveness ratio achieved by each combination of these parameters, ranging from very cost-effective in green (<0.25x Botswana’s annual *per capita* GDP of $8,000) to cost-effective in yellow (0.25–0.5x GDP) and orange (0.5–1x GDP) and not cost-effective in red (>1x GDP). The base case combination (nine-percentage point incremental increase in ART coverage for an incremental CP cost of $1.7 million) is indicated by the ** in the upper left cell. **CP**, combination prevention; **SOC**, standard of care; **ART**, antiretroviral therapy; **ICER**, incremental cost-effectiveness ratio; **QALY**, quality-adjusted life year; **GDP**, gross domestic product.

When we simultaneously varied the incremental cost of the CP intervention and the impact of the program on ART coverage and infections averted, CP remained cost-effective over a wide range of parameters (Figure 4). When the impact of the intervention was two-thirds of the base case, with an increase in ART coverage of six percentage points and 203 infections averted, CP remained cost-effective even when intervention costs were 2.5x the base case. When the impact of the intervention was reduced to one-third of the base case (a three-percentage point increase in ART coverage and 102 infections averted), CP remained cost-effective if the intervention cost did not exceed 1.5x the base case. If the impact of the intervention was only 10% of the base case (a 0.9-percentage point increase in ART coverage and 31 infections averted), the CP intervention would not be cost-effective at base case cost. In this case, costs would need to be 42% lower to meet the threshold of 0.5x GDP per capita per QALY gained. Even with a three-percentage point increase in ART coverage and ~10% reduction in infections, corresponding to the lower bound of the 95% CI for the incidence reduction observed in the trial, the ICER remained below 25% of annual *per capita* GDP in Botswana per QALY gained.

## DISCUSSION

We projected the long-term clinical impact, cost, and cost-effectiveness of a combination HIV prevention intervention implemented in the Botswana Combination Prevention Project. Based on trial outcomes, we estimated 1,418 additional PWH were started on ART and 304 infections were averted due to the combination prevention intervention, compared to the standard of care. The 2,086 QALYs of long-term total health gain produced by CP were split equally between prevalent cases starting earlier on ART and those in whom an infection was averted. Although the CP intervention required a large initial investment—about $1.7 million more than SOC prevention to cover a target population of 55,000—and will generate additional HIV care costs in those already infected who start ART earlier, these costs will likely be largely offset over time due to the prevention of new infections. Indeed, our analysis projects that HIV care costs saved by averting infections will offset nearly all CP program costs and treatment costs for the additional PWH started on ART, resulting in a long-run net cost of $157,000 and an ICER of $79 per QALY.

These results were most dependent on the cost of the CP intervention, the cost of HIV care, and the impact of the intervention on infections averted and ART coverage gained. Within plausible ranges, the CP intervention would remain cost-effective.

This study can also be interpreted in the context of two other major international trials assessing the ‘test and treat’ paradigm. The PopART trial (HPTN 071) in South Africa and Zambia tested two versions of combination prevention and showed that combination prevention improved ART coverage and viral suppression. A decrease in HIV incidence was observed with one version of combination prevention, but not the other. A report of cost-effectiveness suggests that for a comparable scenario the intervention as implemented in the trial had an ICER of $326/disability-adjusted life year (DALY) in South Africa and $258/DALY in Zambia.^26^ The PopART results, though slightly less favorable when comparing base cases, are broadly consistent with our findings regarding the value of combination prevention considering differences in epidemiological context, case-finding and HIV treatment costs, adherence and viral suppression in people on ART, modeled time horizon, as well as parameter and model uncertainty in both analyses. The SEARCH study of over 350,000 people in Kenya and Uganda showed an increase in viral suppression in PWH from 68% to 80% but no change in HIV incidence. Cost-effectiveness results have not yet been reported.^27,28^

Other case-finding approaches such as index contact testing, social network testing and self-testing have also proven cost-effective.^31,32^ These approaches are not intended as alternatives; they complement one another as part of a strategic mix which depends on the context in which they are implemented. While the unit costs of an intensive “saturation” campaign are high relative to facility-based approaches, the testing campaigns led to the identification of nearly all remaining unaware and out of care PWH in the intervention communities, in turn leading to observed incidence reduction within the study period.

This analysis of the BCPP trial has several limitations. We could not evaluate the contribution of each aspect of the CP intervention to the benefits observed in the trial. We did not have HIV care cost data from BCPP and used cost estimates from other studies.^23,29,30^ Further, we only included first-order transmissions over ten years, so we may be underestimating the total number of infections averted over time from the intervention. The incremental cost of the testing campaign and the number of PWH started on ART attributable to the intervention are uncertain. The amount and cost of testing and the number of PWH started on ART in the control arm were not measured in the trial so our estimates of these rely on extrapolations from changes in ART coverage measured in a subset (three of 15 pairs) of the communities in both trial arms. Additionally, our analysis compares the CP intervention to a SOC counterfactual that included a baseline survey with HIV testing in 20% of households. If the SOC had not included the baseline survey, both the incremental cost of CP and the incremental health effect would be higher and the net impact on cost-effectiveness would likely be small. Finally, this analysis, based on the BCPP trial, compared CP to an evolving SOC that was short of universal test and treat. Despite these limitations, sensitivity analysis showed that our cost-effectiveness results were robust to variations in efficacy and cost: we found that at base case effectiveness the intervention would remain cost-effective at 2.5x base case cost. Even if effectiveness was substantially reduced, with only a three-percentage point increase in people starting on ART and a 10% reduction in incident infections (102 infections averted), the CP strategy would remain cost-effective compared to a threshold of 50% of Botswana’s annual *per capita* GDP.

As with any study involving long time horizons, results are subject to uncertainty related to advances in testing, treatment, prevention, or other factors that may impact future transmission dynamics or the outcomes of HIV treatment. Despite these uncertainties, the results of sensitivity analyses increase our confidence that the CP intervention, as implemented in Botswana during the Ya Tsie trial, will be cost-effective. Even with substantially higher testing campaign costs, much smaller increases in ART coverage, and fewer infections averted, the ICER for CP would remain under 50% of Botswana’s annual *per capita* GDP, a reasonable cost-effectiveness threshold for a middle-income sub-Saharan African country.^13,14^ At the time of the study Botswana had, and still has, among the highest pre-existing rates of HIV diagnosis, linkage to ART, and viral load suppression of any country, particularly high HIV-burden countries—and despite this, the CP intervention was cost-effective. It is likely that similar interventions, if they can improve case-finding and provide robust linkage to ART services, would be cost-effective in other settings as well.

## CONCLUSION

Despite high up-front costs, large-scale combination prevention interventions featuring intensive ‘saturation’ testing campaigns that reach PWH not in care and link them to ART are likely to be cost-effective in settings that are comparable to Botswana during the period of the Ya Tsie trial in terms of HIV prevalence, undiagnosed HIV, and care costs. Even in settings with high baseline testing and high ART coverage like Botswana, combination prevention may further improve health among PWH as well as improving population health and lowering HIV care costs by reducing HIV incidence.

## Supplementary Material

Supplement

## Figures and Tables

**Table 1.** Key model parameters

	Base case value		
Input parameter	[Range evaluated]		Reference
Cohort characteristics	Prevalent	Incident	
Sex, female/male, %	68/32	67/33	BCPP
Age, mean (SD), years	37 (11)	35 (11)	BCPP
Initial CD4, mean (SD), cells/μL	449 (266)	569 (226)	BCPP
	[182–715]	[344–795]	
HIV incidence, rate per 100 PY			
*Applies to analysis of lifetime outcomes for those in whom an infection was averted*			
Age, years			BCPP
<18	0.54 [0.25–1.06]		
18–24	0.84 [0.38–1.65]		
25–29	0.77 [0.35–1.51]		
30–39	0.66 [0.30–1.30]		
≥40	0.24 [0.11–0.47]		
Treatment characteristics, 1^st^ line ART, TDF/FTC+DTG	All cohorts		
HIV-1 RNA suppression at 48 weeks, %	98		BCPP
CD4 increase, mean (SD), monthly cells/ μL			^34^
≤2months	106.8 (29.9)		
>2 months	5.3 (1.5)		
Engagement in care	All cohorts		
Probability of loss to follow-up, yearly, %	3.5 [0.4–8.0]		BCPP
Probability of return to care after 12 months, monthly, %	1.0		Assumption
Probability of return to care after OI, one-time, %	50.0		Assumption
Standard of care HIV screening	All cohorts		
Probability of HIV test offer and acceptance, monthly, %	3.0 [0.3–6.0]		BCPP
HIV test characteristics, %			
Sensitivity	100		^35^
Specificity	99		^35^
Probability of linkage to care if positive, one-time, %	86 [50–95]		BCPP
Quality of life (utility weights)	All cohorts		
HIV-uninfected	0.909–0.860		BCPP
HIV-infected			
>500 cells/μL	0.889		
350–500 cells/μL	0.888		
200–349 cells/μL	0.884		
<200 cells/μL	0.879		
CP intervention	All cohorts		
Incremental number of PWH on ART^b^	1,418 [709–2,836]		BCPP
Incremental cost, 2019 USD	1,693,921 [1.0–6.0M]		BCPP

BCPP, Botswana Combination Prevention Project; ART, antiretroviral therapy; TDF/FTC+DTG, tenofovir disoproxil fumarate and emtricitabine with dolutegravir; OI, opportunistic infection; CP, universal test and treat; PWH, people with HIV

a Costs shown here are for drugs only.

b This incremental number of PWH on ART translates to a nine-percentage point increase in ART coverage in the CP arm compared to SOC.

**Table 2.** Clinical and economic outcomes of the CP intervention compared to SOC

	Incident HIV infections^a^	Total life years [discounted]^b^	Total quality-adjusted life years [discounted]^b^	Total cost, $ [discounted]^b,c^	ICER $/QALY
Long-term impact of BCPP Trial					
SOC	972	23,586	20,803	24,124,000	
CP	668	25,847	22,804	24,282,000	
*Difference*	−304	2,261	2,001	157,000	$79

SOC, standard of care; CP, combination prevention; LY, life year; QALY, quality-adjusted life year.

a Infections include those during the trial period, measured in the trial, plus additional first-order transmissions over ten years estimated with the CEPAC model for the cohort that started ART earlier due to the intervention, and its counterfactual.

b Discounted at 3% per year.

c Total costs are rounded to the nearest $100.

## Data Availability

The fully de-identified patient dataset from the BCPP trial is available under certain use restrictions at https://data.cdc.gov/Global-Health/Botswana-Combination-Prevention-Project-BCPP-Publi/qcw5-4m9q and is provided by the US Centers for Disease Control and Prevention. The study protocol is available at https://clinicaltrials.gov/ct2/show/NCT01965470. For collaboration with the CEPAC model please contact the CEPAC team at https://mpec.massgeneral.org/cepac-model/.
