# Supplementary material for: Health impact and cost-effectiveness of HIV testing, linkage, and early antiretroviral treatment in the Botswana Combination Prevention Project
Source: J Acquir Immune Defic Syndr. Author manuscript; Available in PMC 2022 Aug 1. (PMC9295776; doi:10.1097/QAI.0000000000002996)
Supplement: Supplement [file NIHMS1807557-supplement-Supplement.docx]

**Health impact and cost-effectiveness of HIV testing, linkage, and early antiretroviral treatment in the Botswana Combination Prevention Project: Supplement**

Stephen C. Resch, PhD^1^, Julia H. A. Foote, BA^2^, Kathleen E. Wirth, ScD^3,4^, Arielle Lasry, PhD^5^, Justine A. Scott, MPH^2^, Janet Moore, PhD^5^, Fatma M. Shebl, PhD^2,6^, Tendani Gaolathe, MD^7^, Mary K. Feser, BA^2^, Refeletswe Lebelonyane, MD^8^, Emily P. Hyle, MD^2,6,9,10,12,13^, Mompati O. Mmalane, MD^7^, Pamela Bachanas, PhD^5^, Liyang Yu, MS^2^, Joseph M. Makhema, MD^7^, Molly Pretorius Holme, MS^4^, Max Essex, PhD^4,7^, Mary Grace Alwano, MPH^11^, Shahin Lockman, MD^4,6,7,12^, Kenneth A. Freedberg, MD^1,2,6,9,10,13^

**Table of Contents**

**Figure S1.** Analytic overview: cost-effectiveness of the Botswana Combination Prevention Project.

**Methods:** Additional Information

**Table S1.** Additional base case parameters for the CEPAC analysis

**Table S2.**  Additional costs applied outside CEPAC model

**Table S3.**  CEPAC results for PWH started on ART due to the CP intervention and SOC counterfactual.

**Table S4.** CEPAC results for persons in whom an infection is averted due to CP compared to SOC counterfactual in which infection is not averted

**Table S5.** CEPAC model results: first order transmissions per PWH by year with and without early ART start

**Table S6.**  Scaling of observed infections averted in BCPP incidence cohort

**Table S7.** Total infections averted: CP vs. SOC

**Figure S1.** Analytic overview: cost-effectiveness of the Botswana Combination Prevention Project.

This figure outlines the CEPAC model runs used in the cost-effectiveness analysis of the Botswana Combination Prevention Project.

0. Incidence Cohort: To determine the impact per transmission, two sub-cohorts were run (*Acute HIV* and *Averted Infection*). The *Acute HIV* cohort represents those participants that acquired HIV during the BCPP trial, while the counterfactual *Averted Infection* cohort represents those same participants but as if they did not acquire HIV during the trial (however, they may acquire HIV at a later time). Model outcomes from the incidence cohort include discounted quality-adjusted life years and costs and can be found in Table S4. The lifetime difference in discounted quality-adjusted life-years and costs between the *Acute HIV* and *Averted Infection* cohorts represents the negative health impact (QALY_Tx_) and additional costs (Cost_Tx_) per transmission at a point in time, respectively. QALY_Tx_ and Cost_Tx_ are then applied to the prevalent cohort model outcomes.

1. Prevalent Cohort: To account for the benefit of earlier detection and ART start, two sub-cohorts were run (CP and SOC). The CP cohort represets PWH who were detected and started on first-line ART during the BCPP trial; this cohort reflects the characteristics of the BCPP participants with HIV who were undetected or unlinked to care prior to the trial. The counterfactual SOC cohort represents those same PWH but as if they were not detected or linked to care during the trial (however, they may be linked to care through standard of care testing at a later time). Model outcomes from the prevalent cohort include first-order transmissions over ten years and discounted quality-adjusted life years and costs. Model outcomes among prevalent PWH excluding the impact of transmissions (QALY_prevalent, without Tx_ and Cost_prevalent, without Tx_) are shown in blue and can be found in Table S3. We then multiply the impact per transmission (QALY_Tx_ and Cost_Tx_) by the number of first-order transmissions and add the product to the discounted quality-adjusted life years (QALY_prevalent, without Tx_) and costs (Cost_prevalent, without Tx_) for each sub-cohort. The sum then represents the total quality-adjusted life years (QALY_prevalent, with Tx_) and costs (Cost_prevalent, with Tx_), including the impact of transmissions. Model outcomes among prevalent PWH including the impact of transmissions are shown in green and can be found in Table 2.

**QALY**, quality-adjusted life-years; **Tx**, transmission; **ART**, antiretroviral therapy; **CP**, combination prevention; **PWH**, people with HIV; **SOC**, standard of care.

**Figure S1.** Analytic overview: cost-effectiveness of the Botswana Combination Prevention Project


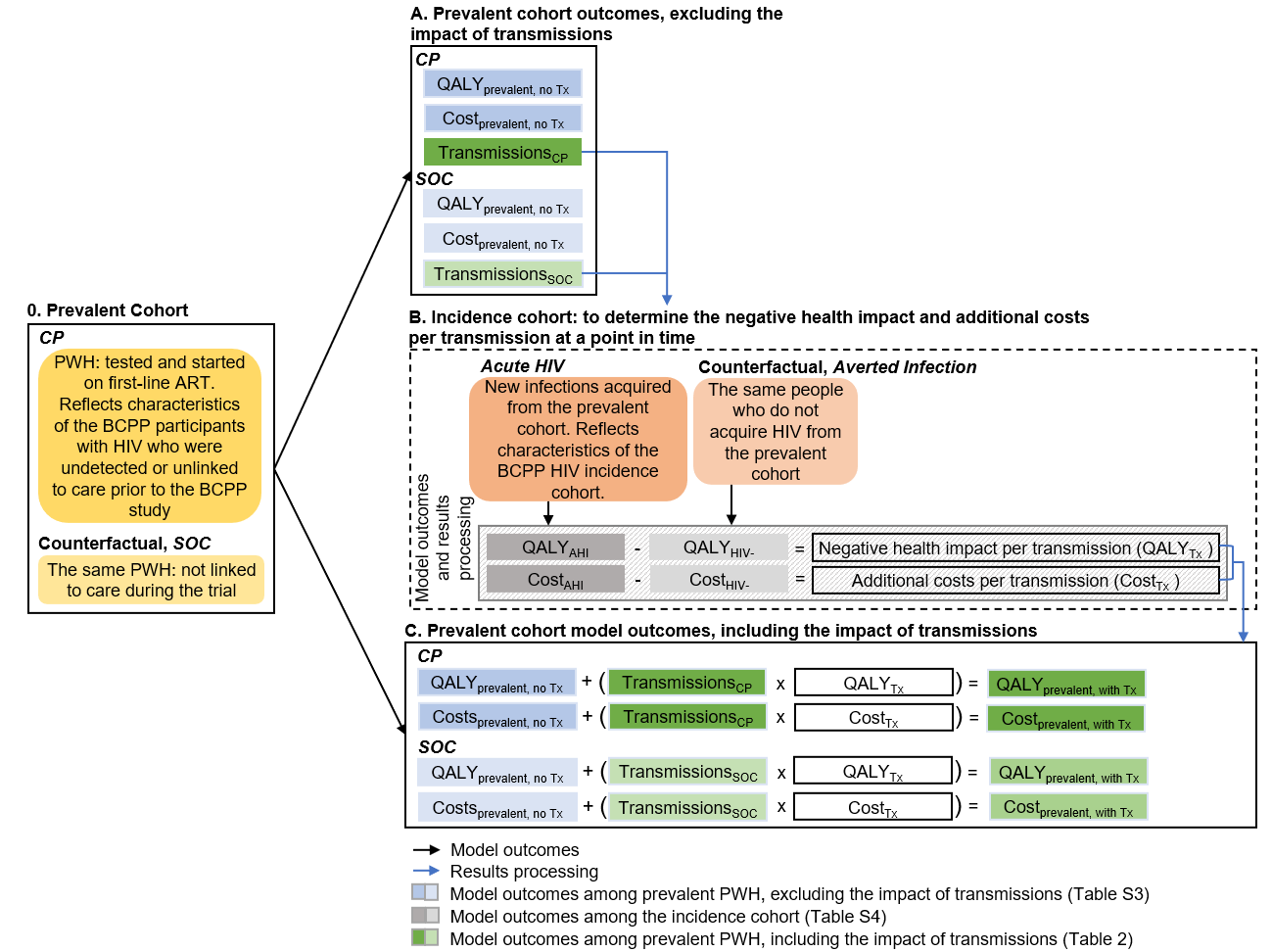


**Methods: Additional Information**

**Calculation of Averted Infections after Trial Period Due to Early ART in PWH**

A cohort of PWH matching characteristics of those detected by the CP intervention was modeled with CEPAC to estimate how many first-order transmissions were prevented by early ART start. In the analysis, the model was run once with the cohort put on ART at model start, and a second time (representing the counterfactual) in which they begin the model with their HIV undetected. Table S5 shows the results. For each PWH started on ART, the model estimates that approximately 0.11 transmissions would be prevented over 10 years. Most of the effect occurs in earlier years because in the counterfactual many PWH eventually become detected and start on ART. From month 30 onward, 0.03 transmissions are averted per PWH started early on ART, resulting in a total of 42 additional averted infections.

For the first 29 months (i.e., the trial period), we used the prevention effect observed in the trial period, which may be attributable not only to increased case-finding and linkage but also to VMMC and differences in ART eligibility criteria. The infections observed in the HIV incidence cohort (HIC), over an average of 29 months follow-up time, were scaled to the HIV-negative population size in the intervention communities of the trial, as shown in Table S6. We calculated the total infections in the control arm, and then applied the 0.69 incidence ratio observed by permutation test in the analysis of trial results [9]. This results in an estimate of 262 infections averted. To this, we added model-based estimates of the additional first order transmissions that would be prevented after the trial period due to the additional PWH started on ART. From month 30 to 120, an additional 42 infections are prevented due to 1,418 PWH that were started on ART because of the CP intervention, for a total of 304 infections averted.

We may underestimate the full effect of the CP intervention because the model-based extension of the time horizon can only capture the first-order effect of additional ART starts. While we would expect no further effect of difference in ART eligibility criteria due to the move to universal eligibility as the national standard, the effect of the VMMC intervention after the trial period and the dynamic effects of current incidence on future incidence were not captured in our model.

**Modeling: Additional Details**

To increase the precision of per-person health benefits and cost, we simulated cohorts of 1 million persons. We then scaled outcomes to the population size of the intervention communities in the trial.

**ART Eligibility**

ART eligibility in both arms changed over the duration of the trial. In the CP arm, ART was available for adults with CD4 ≤350 cells/μL (moving to ≤500 cells/μL in 2015) and/or viral load ≥10,000 copies/mL. In the SOC arm, only adults with CD4 ≤350 cells/μL were eligible for ART. In June 2016, universal ART became standard of care in Botswana and was adopted in both arms. Adults with World Health Organization (WHO) stage 3 or 4 disease and pregnant or breastfeeding women in both arms were eligible for ART throughout.

**Calculation of Incremental ART initiations and Background Testing Level**

In the trial’s CP arm, 3,065 PWH were started on ART: 455 via the baseline survey and 2,610 via the testing campaign. Accounting for observed differences in linkage between the arms, we estimated that the baseline survey in the control arm resulted in 388 ART initiations.

Indicators measured in a subset of three control and three intervention communities at baseline (random ~20% sample) and study completion (remaining ~80% of households) found an increase in ART coverage of 19 percentage points in intervention communities, compared to a ten percentage point increase in control communities. Accounting for observed loss-to-follow-up found that a nine percentage point difference in the increase in ART coverage would have required 1418 incremental ART initiations in the CP arm.

The BCPP trial did not collect data on background testing in either arm. To estimate the level of background testing in each arm, we used the percentage change in ART coverage during the trial to infer the total case-finding in each arm as described above. Then, we accounted for the contribution of case-finding through trial activities (campaigns and baseline survey) and for loss-to-follow-up from ART, both from trial data. We assumed that ‘pre-ART’ PWH in care were started on ART in both arms by trial completion due to Botswana’s switch to universal eligibility. We attributed any additional ART initiations to background testing, totaling 350 in CP and 1604 in SOC.

**Adherence**

Participants were considered adherent if their self-reported ability to take all their medications in the past four weeks was “very good.” Of participants, 82% reported “very good” adherence; among this group virologic suppression at 48 weeks was 99%. Even among those with lower self-reported adherence (18% of participants), suppression at 48 weeks was 95%.

**Quality of Life**

Quality of life weights were from the EuroQol EQ-5D-5L incorporated into the baseline survey and were scored using a previously developed function for a representative population of Zimbabwe, which is the only EQ-5D scoring function available for sub-Saharan Africa. We applied these quality-of-life adjustments in CEPAC, stratified by HIV status and CD4 count.

**Costing: Additional Details**

Testing campaign costs were obtained from a costing study in 11 of the 15 intervention communities and scaled to the trial size.^1^

Linkage counselor cost was based on BCPP trial administrative data; each community was assigned a cost of $13,110, which supported 50% of a full-time linkage coordinator and 30% of three full-time HIV community counselor salaries (Table S2).

For background testing, we estimated the testing level that would have had to occur to reach observed ART coverage levels. We assumed the yield and linkage rates for background testing matched the testing campaigns but that the cost was $11.15 per person tested, based on a previous systematic review.^2^ VMMC costs were estimated by extrapolating the number of incremental procedures implied by the change in VMMC coverage and multiplying by $116 per procedure.^3^

**References: Supplement**

1. Lasry A, Bachanas P, Suraratdecha C, et al. Cost of community-based HIV testing activities to reach saturation in Botswana. AIDS Behav. 2019 Apr;23(4):875–82.

2. Sharma M, Ying R, Tarr G, et al. Systematic review and meta-analysis of community and facility-based HIV testing to address linkage to care gaps in sub-Saharan Africa. Nature. 2015 Dec;528(7580):S77–85.

3. Pineda-Antunez C, Martinez-Silva G, Cerecero-Garcia D, et al. Meta-analysis of average costs of HIV testing and counselling and voluntary medical male circumcision across thirteen countries. Afr J AIDS Res. 2019 Dec;18(4):341–9.

4. Attia S, Egger M, Muller M, et al. Sexual transmission of HIV according to viral load and antiretroviral therapy: systematic review and meta-analysis. AIDS. 2009 Jul 17;23(11):1397–404.

5. Wawer MJ, Gray RH, Sewankambo NK, et al. Rates of HIV-1 transmission per coital act, by stage of HIV-1 infection, in Rakai, Uganda. J Infect Dis. 2005 May 1;191(9):1403–9.

6. United Nations. World population prospects: Botswana [Internet]. 2019 [cited 2020 Jan 29]. Available from: https://population.un.org/wpp/Download/Standard/Population/

7. World Health Organization. Global health estimates 2016: disease burden by cause, age, sex, by country and by region, 2000-2016. Geneva, Switzerland; 2018.

8. Gaolathe T, Wirth KE, Pretorius Holme M, et al. Botswana’s progress toward achieving the 2020 UNAIDS 90-90-90 antiretroviral therapy and virological suppression goals: a population-based survey. Lancet HIV. 2016 May;3(5):e221-30.

9. Makhema J, Wirth KE, Pretorius Holme M, et al. Universal testing, expanded treatment, and incidence of HIV infection in Botswana. N Engl J Med. 2019 Jul 18;381(3):230–42.

| Input parameter | Base case value | | Reference |
| --- | --- | --- | --- |
| Cohort characteristics |  | |  |
| Plasma HIV-1 RNA distribution, copies/mL, % | Prevalent | Incident | BCPP |
| >100,000 | 16 | 2 |  |
| 30,001-100,000 | 20 | 6 |  |
| 10,001-30,000 | 19 | 8 |  |
| 3,001-10,000 | 17 | 27 |  |
| 501-3,000 | 14 | 18 |  |
| <500 | 14 | 39 |  |
| Transmission rates, per 100 PY  *Applies to analysis of additional infections averted after trial period due to earlier ART in prevalent cohort* | | | |
| Acute infection | 62.56 [31.28-125.12] | | ^4,5^ |
| Chronic infection, copies/mL |  | |  |
| >100,000 | 9.03 [4.51-18.06] | | ^4,5^ |
| 30,001-100,000 | 8.12 [4.06-16.24] | |  |
| 10,001-30,000 | 8.12 [4.06-16.24] | |  |
| 3,001-10,000 | 4.17 [2.09-8.34] | |  |
| 501-3,000 | 2.06 [1.03-4.12] | |  |
| 20-500 | 2.06 [1.03-4.12] | |  |
| <20 | 0.00 [0.00-0.00] | |  |
| Natural history | Both cohorts | |  |
| Probability of non-AIDS-related mortality (range by age), monthly, % |  | | ^6,7^ |
| ≤ 14 years | 0.003-0.281 | |  |
| 15-24 years | 0.005-0.014 | |  |
| 25-39 years | 0.009-0.020 | |  |
| 40-49 years | 0.020-0.046 | |  |
| 50-59 years | 0.046-0.118 | |  |
| ≥ 60 years | 0.114-2.096 | |  |
| Probability of AIDS-related mortality off ART (range by CD4 count and history of prior OI), monthly, % | 0.035-1.088 | | BCPP, ^8^ |
| Probability of AIDS-related mortality on ART (range by CD4 count and history of prior OI), monthly, % | 0.001-0.036 | | BCPP, ^8^ |

**Table S1.** Additional base case parameters for the CEPAC analysis

**BCPP**, Botswana Combination Prevention Project; **PY**, person years; **ART**, antiretroviral therapy; **OI**, opportunistic infection

**Table S2.** Additional costs applied outside CEPAC model

| Costs, 2019 USD | Base case value  [Range evaluated] | Reference |
| --- | --- | --- |
| Testing and intervention costs |  |  |
| CP intervention (testing campaign and baseline survey), per person tested | 32.76 | ^1^ |
| Background HIV testing, per test | 11.15 |  |
| Voluntary male medical circumcision (VMMC), per procedure | 116 | ^3^ |
| Linkage counseling |  | ^a^ |
| Linkage coordinator salary, 1 per community at 50% time | 6,628 |  |
| HIV community counselor salary, 3 per community at 30% time | 6,482 |  |
| Laboratory monitoring, per test |  |  |
| CD4 test | 13.25 [6.63-26.50] |  |
| HIV RNA test | 24.76 [12.38-49.52] |  |
| Treatment costs, yearly ^a^ |  |  |
| 1^st^ line ART, TDF/FTC+DTG | 77.76 [38.88-155.52] |  |
| 2^nd^ line ART, AZT+3TC+ LPV/r | 288.36 [144.18-576.72] |  |
| Co-trimoxazole | 10.32 |  |
| Routine HIV care, monthly |  |  |
| >500 cells/µL | 19.38 |  |
| 350-500 cells/µL | 25.81 |  |
| 200-349 cells/µL | 31.08 |  |
| 50-199 cells/µL | 67.66 |  |
| <50 cells/µL | 154.56 |  |

**USD**, United States dollar; **CP**, combination prevention; **ART**, antiretroviral therapy; **TDF/FTC+DTG**, tenofovir disoproxil fumarate and emtricitabine with dolutegravir; **AZT+3TC+LPV/r**, zidovudine with lamivudine and lopinavir/ritonavir

^a^ In an email from A. Lasry (ftn9@cdc.gov) on March 12, 2019.

**Table S3.** CEPAC results for PWH started on ART due to the CP intervention and SOC counterfactual

|  | Total per prevalent PWH started early on ART | | | | | | | | |
| --- | --- | --- | --- | --- | --- | --- | --- | --- | --- |
|  | Undiscounted | | | |  | | Discounted | | |
|  | LYs | QALYs | HIV-related care costs ($) |  | | LYs | | QALYs | HIV-related care costs ($) |
| SOC | 31.18 | 27.46 | 17,515 |  | | 18.47 | | 16.30 | 10,209 |
| CP | 33.01 | 29.07 | 18,816 |  | | 19.49 | | 17.20 | 11,078 |
| Difference | 1.83 | 1.61 | 1,301 |  | | 1.02 | | 0.90 | 869 |

**PWH**, person living with HIV; **ART**, antiretroviral therapy; **CP**, combination prevention; **SOC**, standard of care; **LY**, life year; **QALY**, quality-adjusted life year

**Table S4.** CEPAC results for persons in whom an infection is averted due to CP compared to SOC counterfactual in which infection is not averted

|  | Total per averted infection | | | | | | |
| --- | --- | --- | --- | --- | --- | --- | --- |
|  | Undiscounted | | |  | Discounted | | |
|  | LYs | QALYs | HIV-related care costs ($) |  | LYs | QALYs | HIV-related care costs ($) |
| New infection | 33.10 | 29.17 | 17,636 |  | 19.28 | 17.03 | 9,967 |
| Averted infection^a^ | 39.22 | 34.57 | 1,657 |  | 22.01 | 19.46 | 728 |
| Difference | 6.12 | 5.41 | -15,979 |  | 2.74 | 2.43 | -9,239 |

**CP**, combination prevention; **LY**, life year; **QALY**, quality-adjusted life year

^a^ Averted infection includes: 1) individuals in whom an infection was averted during the trial and who did not acquire HIV in the 10-year follow-up period, and 2) those in whom an infection was averted during the trial but who acquired HIV after the trial based on a monthly probability of acquiring HIV.

|  | First order transmissions per year from PWH | | |
| --- | --- | --- | --- |
| Year | SOC | CP | Difference |
| 1 | 0.051 | 0.007 | 0.044 |
| 2 | 0.035 | 0.003 | 0.032 |
| 3 | 0.024 | 0.005 | 0.019 |
| 4 | 0.017 | 0.006 | 0.011 |
| 5 | 0.013 | 0.007 | 0.006 |
| 6 | 0.010 | 0.008 | 0.002 |
| 7 | 0.009 | 0.008 | 0.001 |
| 8 | 0.009 | 0.009 | 0 |
| 9 | 0.009 | 0.009 | 0 |
| 10 | 0.009 | 0.009 | 0 |
| **Total** | **0.186** | **0.071** | **0.115** |

**Table S5.** CEPAC model results: first order transmissions per PWH by year with and without early ART start

**PWH**, person living with HIV; **ART**, antiretroviral therapy; **SOC**, standard of care; **CP**, combination prevention

**Table S6.** Scaling of observed infections averted in BCPP incidence cohort

|  | SOC | CP |
| --- | --- | --- |
| HIV incidence cohort (HIC) size^a^ | 4,292 |  |
| Person-years of follow-up | 9,802 |  |
| New infections observed in HIC | 90 |  |
| Annualized incidence | 0.0092 |  |
| Relative incidence by permutation test |  | 0.69 |
| Trial population | 54,674 |  |
| Estimated PWH at start of trial | 14,270 |  |
| HIV- population | 40,404 |  |
| Person-years | 92,274 |  |
| New infections scaled to HIV- population | 847 | 585 |

**BCPP**, Botswana Combination Prevention Project; **SOC**, standard of care; **CP**, combination prevention; **PWH**, person living with HIV

^a^Number of participants in the HIV incidence cohort for whom person-time was reported.

**Table S7.** Total infections averted: CP vs. SOC

|  | New infections during the trial, as measured in HIC cohort | Additional first order transmissions over 10 years from PWH started on ART | Total transmissions over 10 years |
| --- | --- | --- | --- |
| SOC | 847 | 125 | 972 |
| CP | 585 | 83 | 668 |
| Difference | 262 | 42 | 304 |

**CP**, combination prevention; **SOC**, standard of care; **HIC**, HIV incidence cohort; **PWH**, person living with HIV; **ART**, antiretroviral therapy
